# Chitosan Nanoparticles for Enhanced Delivery of *Sida cordifolia* Extract: Formulation, Optimization and Bioactivity Assessment

**DOI:** 10.3390/ph16111561

**Published:** 2023-11-06

**Authors:** Perwez Alam, Mohd Imran, Shahnawaz Ahmed, Haya Majid, Ali Akhtar

**Affiliations:** 1 Department of Pharmacognosy, College of Pharmacy, King Saud University, P.O. Box 2457, Riyadh 11451, Saudi Arabia; aakhtar@ksu.edu.sa; 2 Department of Pharmacognosy, School of Pharmaceutical Education and Research, Jamia Hamdard, New Delhi 110062, India; imranidrisi00786@gmail.com; 3 Department of Clinical Research, Max Super Speciality Hospital, Saket, New Delhi 110017, India; shahnawazahmed7300@gmail.com; 4 Department of Translational and Clinical Research, School of Chemical and Life Sciences, Jamia Hamdard, New Delhi 110062, India; hayamajid1.hm@gmail.com

**Keywords:** *Sida cordifolia* hydroalcoholic extract, chitosan, nanoparticles, optimization, CLSM, Box–Behnken design

## Abstract

In a continuous search for an essential antidiabetic agent, Sida cordifolia hydroalcoholic (SCHA) extract-loaded chitosan nanoparticles (SCHA-CS-NP) were optimized. The Box–Behnken design (BBD Design-Expert software, version 14) with three parameters was used to optimize the nanoparticles after creating them using the ion gelation method. The chitosan and Tween 20 contents and the stirring speed were chosen as the independent variables, and their separate and combined effects on particle size (Y1), polydispersity index (Y2) and entrapment efficiency (Y3) were observed. The optimized formulation showed a particle size of 51 nm, an entrapment efficiency of 84.54% and a polydispersity index of 0.391. Physicochemical characterization, Fourier transform infrared spectroscopy (FTIR), differential scanning calorimetry (DSC), a drug release study, an ex vivo permeation study, and an antioxidant study were performed. Confocal laser scanning microscopy (CLSM) images demonstrated that chitosan nanoparticles loaded with rhodamine B-laden SCHA extract had superior penetration compared to the control (rhodamine B solution). Furthermore, compared to conventional ascorbic acid (IC_50_ = 45 µg/mL), a superior antioxidant activity was discovered for SCHA-CS-NPs (IC_50_ = 86.45 ± 2.24 µg/mL), while SCHA-CS-NPs also exhibited strong antidiabetic potential (IC_50_ = 93.71 ± 1.79 µg/mL) compared to standard acarbose (IC_50_ = 97.25 ± 1.43 µg/mL). The overall results demonstrated that SCHA-CS-NPs are a promising and efficient formulation for oral delivery.

## 1. Introduction

Diabetes is a major medical problem affecting people globally. It is caused by persistently high blood glucose levels, resulting in the inability of beta cells (β cells) in the pancreas to make adequate insulin or the inability of cells in the body to use insulin. Type 1 and 2 diabetes are the two most common types of diabetes. The International Diabetes Federation estimated that 537 million people worldwide had diabetes in 2021. This number is expected to increase in high income and low- to middle-income countries. The cases of diabetes are expected to increase by 629 million by the end of 2045 if proper administration and control are not implemented. Type 2 diabetes (T2D) accounts for more than 90% of all diabetes cases, due to low insulin activity (insulin resistance) and slowed insulin production by pancreatic intestine cells. Glucose levels in the blood are elevated by this disorder [1]. Despite a broad understanding of this disease at the subatomic level and the revelation of new medications, diabetes and its symptoms remain generally untreated. 

Natural vegetation has long been a rich hotspot for remedial specialists, particularly against diabetes [2]. More than 800 plants have antidiabetic properties. *Sida cordifolia*, a bush from the Malvaceae family, has proven efficacy in diabetes treatment [3]. Its therapeutic role has been evidenced through its leaves, stems and seeds, which are used for their antirheumatic, antipyretic, asthmatic, purgative, diuretic, vasorelaxant, hypotensive, CNS depressant, cancer prevention and hypoglycemic effects [4]. It is a medicinal plant with a wide range of uses due to its high phytochemical content. The anti-inflammatory, analgesic and antioxidant characteristics of *Sida cordifolia* have led to its use in various traditional herbal medicines. The hypoglycemic activity of *Sida cordifolia* varies based on the sections of the plant, dosage and type of extract used. Its aqueous extract has been found to contain reducing sugars, glycosides, resins, alkaloids, flavonoids, saponins and sterols [5,6]. Its anti-diabetic properties are useful for improving immune function and accelerating wound healing. Traditional and contemporary herbal medicine practitioners are interested in *Sida cordifolia* due to its diverse range of phytochemicals and biological activity [7].

With its potential for controlled release and to protect active components from enzymatic or environmental degradation and localized retention, nanoparticle technology has emerged as a leading drug delivery method. The methods of formulating nanoparticles can be easily scaled up and used to create a wide variety of pharmaceuticals. Because they are biodegradable, biocompatible and have widely accessible formulation techniques, polymeric nanoparticles have gained significant importance among various classes of nanoparticles. Additionally, their range of applications has expanded to include various chemical drug classes and dosage forms. Due to their low toxicity and adaptable physical properties, chitosan-based nanoparticles (NPs) are particularly suitable for drug administration. Chitosan NPs are used to treat eye infections, cancer, gastrointestinal disorders, pulmonary disorders and cancer. The growing understanding of chitosan characteristics and chemical or physical modification techniques, which are used for the optimization of nanoparticle drug loading and release features, is the foundation for recent research on chitosan-based NPs for non-parenteral drug administration [8]. Chitosan-based nanoparticles can be used in several administration routes to distribute active ingredients, such as pharmaceuticals or natural products, including oral and parenteral delivery. Since chitosan NPs combine the properties of polymers with a tunable size and the possibility of customizable surface modification, they offer a very promising and adaptable means to overcome the bioavailability and dependability issues of most dynamic fixings. 

Deacetylation is the process used to convert chitin into chitosan. A strong solution of sodium hydroxide (25–50%) at a high temperature (90–120 °C) can be used in a chemical process, or a deacetylase-based bioprocess can be used [9]. The primary objective of this study was to assess the potential antidiabetic effects of *Sida cordifolia* hydroalcoholic (SCHA) extract derived from the aerial component of the plant when formulated into chitosan NPs. The NPs were thoroughly characterized using various techniques, including Fourier transform infrared spectroscopy (FTIR) for structural analysis, confocal laser scanning microscopy (CLSM) for visualization and zeta size and potential measurements. Additionally, the entrapment efficiency (EE) was determined through ultracentrifugation, while the drug release kinetics were investigated using dynamic dialysis. Transmission electron microscopy (TEM) was employed to directly assess NP size and morphology, while differential scanning calorimetry (DSC) was used to evaluate the physical properties and reliability of the nanoparticles. In vitro release studies were conducted to understand their release profiles, and stability studies were conducted to determine the shelf life of multidose formulations. Furthermore, their antioxidant properties were evaluated using the 2, 2-diphenyl-1-picrylhydrazyl DPPH assay, and the potential antidiabetic activity was assessed using the α-amylase assay. This comprehensive approach aims to reveal the therapeutic potential of SCHA extract and chitosan-loaded SCHA NPs in managing diabetes.

## 2. Results and Discussion

### 2.1. Optimization of Sida cordifolia-Loaded Chitosan NP

According to the Box–Behnken design (BBD) software, the effect of independent variables such as the concentrations of chitosan (X1) and Tween 20 (X2) and the stirring speed (X3) were assessed for their individual and combined effects on particle size (Y1), polydispersity index (Y2) and EE (Y3). Seventeen detailing runs with five focuses were assessed for outcome errors (Table 1). The polynomial condition of the formulation was likewise used to decipher the results. The negative and positive signs of the condition addressed the positive and adverse impacts on the particle size and viability of exemplification. The outcome of the free factors was further fitted to several motor models, including direct, 2 F1 and quadratic, to assess their impact.

#### 2.1.1. Effects of Independent Variables on Particle Size (Response Y1)

**Figure 1:**
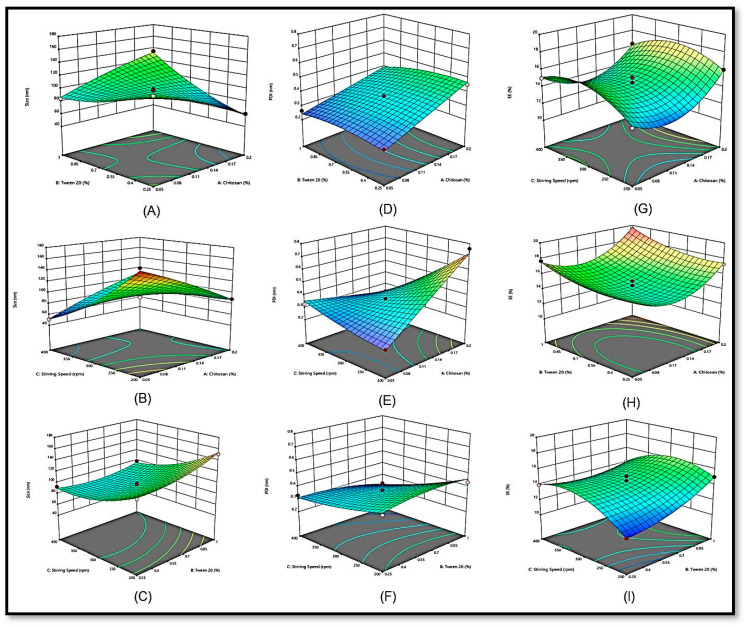
3D response surface plot showing the effect of independent variables on particle size (Y1) (**A**–**C**), polydispersity index (Y2) (**D**–**F**) and entrapment efficiency (Y3) (**G**–**I**).

As per the runs depicted by the response surface methodology, the largest size of the SCHA extract-loaded chitosan nanoparticles (SCHA-CS-NPs) was 151 nm, while the smallest NP had a diameter of 51 nm (Table 2). Chitosan (0.05 mg), Tween 20 (0.625 mL), and stirring speed (400 rpm) were used to develop the base size of 51 nm (F10), while chitosan (0.125 mg), Tween 20 (1 mL), and stirring speed (200 rpm) were used to create the largest size of 151 nm (F2). The 3D response surface (Figure 1) also depicted the effects of autonomous elements. According to the findings, the particle size decreased as the stirring speed increased. The particle size was increased by raising the chitosan content from 0.05 to 0.125 mg. A large particle size was obtained by using chitosan (0.125 mg) and Tween 20 (1 mL) and limiting the stirring speed. For formulations F1 and F4, the same effect of chitosan was observed; a smaller amount of chitosan determined the basic particle size. The particle size was greater in formulations F2 and F6, which had the highest chitosan concentrations. The size of preparation F8 was 93 nm, which increased to 97.09 nm (F9) when the Tween 20 concentration was increased to 0.625 from 0.25 mL. The stirring speed (X3) had a more noticeable impact on particle size. The particle size drastically decreased as the stirring period increased. Comparing the formulations (F2 and F17) prepared with the shortest stirring speed (200 rpm) to formulations F3 and F10 (400 rpm) indicated that the former exhibited a larger size (Table 2). As the stirring speed was extended, a small reduction in particle size was observed in formulation F10 (51 nm) compared to formulation F2 (151 nm). Quadratic models with an F value of 89.70 were observed in the polynomial condition. The importance of the model words was not entirely resolved by the *p*-value, which was less than 0.0500, and the clatter likelihood, which was assessed as 18.96%. With the model terms, both the individual and combined components were significant (X1, X2, X3, X1X2, X1X3, X2X3, X12 and X32). The estimated association coefficient (R^2^) of 0.9914 supports the best-fit model. The authentic and normal values of the association coefficient were 0.9804 and 0.9046, respectively (Table 3). The model’s strength was demonstrated by the closeness of the true and expected values. The model may be used to assist with particle estimation, since the indicator could be calculated with adequate accuracy and was obtained as 35.667.

Y1 = 94.84 − 4.59 X1 + 9.21 X2 − 22.52 X3 + 27.28 X1X2 + 33.99 X1X3 − 6.48 X2X3 − 3.44 X1^2^ + 8.56 X2^2^ + 12.12 X3^2^

#### 2.1.2. Effects of Independent Variables on the Polydispersity Index (Y2)

According to the polydispersity index profiles of the NP formulations, formulation (F7) exhibited the highest polydispersity index (0.761). The variables chitosan (0.2 mg), Tween 20 (0.625%), and stirring speed (200 rpm) were used to create the formulation. For formulation F13, a minimum polydispersity index (PDI) of 0.216 was found. The chitosan NPs (0.05 mg), Tween 20 (0.625%), and stirring speed (200 rpm) demonstrated a minimal polydispersity index. The PDI produced by the prepared NPs differed significantly. The 3D response surface graphic shows the impact of the independent variables.

The presence of Tween 20 helped improve the PDI and enabled the minimum polydispersity. Compared to traditional NPs, the capacity to maintain particle integrity by employing Tween 20 was significant. Upon increasing the chitosan content (X1) in the formulations, a higher polydispersity index for the SCHA extract was observed in the 3D response surface graph (Figure 1). However, because chitosan resembles biological membranes, it facilitates the dispersion of drugs through cell membranes. At a maximum dose of chitosan (0.2 mg), formulations F7 and F6 had a greater polydispersity index profile than formulations F2 and F17, which contained less chitosan (0.125 mg). Additionally, as the stirring period was prolonged, the EE of the SCHA extract increased. The polydispersity index was directly proportional to the stirring speed [10].

In comparison to formulation F4 (stirring speed 300 rpm), which showed a PDI of 0.359 with fixed chitosan (X1) and Tween 20, F11 (stirring speed 300 rpm) generated a higher PDI of 0.371 (X2). It is logical to assume that as the stirring speed duration is prolonged, the particle size is reduced, and the PDI increases. In this study, Tween 20 and PDI showed a reverse association. As Tween 20 alters the chitosan bilayers, which offer stiffness to decrease the PDI of the NPs, it increases the size of the chitosan particles.

The effects of the variables X1, X2, X3, X1X2, X1X3 and X2X3 were shown to be significant both individually and together. The results of quadratic impacts were visible in the polynomial equation. The F value was 55.08, and the *p*-value was less than 0.0500. Noise was minimal (2.41%). The correlation coefficient R^2^ was 0.9822, indicating that the model fitted the data well. The expected R^2^ of 0.8371 and the adjusted R2 of 0.9643 were approximately equivalent (Table 3). The model can be used to facilitate the design of the PDI from the particles, because the signal-to-noise ratio could be determined with sufficient accuracy and was 29.532.

Y2 = 0.3612 + 0.0984 X1 − 0.0155 X2 − 0.0886 X3 − 0.0208 X1X2 − 0.1600 X1X3 − 0.0323 X2 X3BC − 0.0274 X2^2^ + 0.0184 X3^2^

#### 2.1.3. Effects of Independent Variables on the EE (Y3)

The highest and lowest EEs were 84.54% for F6 and 55.90% for F13. The 3D response surface represented the effect of free factors (Figure 1). According to the findings, chitosan (X1) may impact the EE. The NP fixation increased from 0.05 to 0.125 mg. By maintaining constant values of Tween 20 (X2) and stirring speed (X3), the entanglement (E) proficiency increased significantly from 80.07% (F15) to 84.54% (F6).

Compared to formulations F5, F10, F13 and F16, which contained the lowest chitosan concentrations, formulations F3, F6, F12 and F15 demonstrated a better EE of SCHA-CS-NPs. This demonstrated that when the Tween 20 concentration increased, the %EE of the NPs increased. The lipophilic properties of the SCHA extract, which exhibit greater trapping inside the chitosan NPs, also substantially impacted the EE. The effectiveness of entanglement (EE) shows an inverse correlation with the stirring speed. As the stirring speed increases, the productivity of entanglement steadily declines. One significant challenge in decreasing particle size and effectively transporting particles to their desired location is the use of extremely high stirring speeds, referred to as ultrastirring speeds. Nonetheless, this intensive stirring process also impacts the retention of pharmaceuticals by causing particle rupture, which in turn enhances the release of medications from the particles and diminishes their entrapment within the particles (Table 2 and Table 3) [11].

Formulation F11 had a lower EE (72%) and a longer stirring speed (300 rpm) than formulation F2 (74%), which had a shorter stirring speed (20 rpm). The proficiency in entanglement was substantially impacted by Tween 20. Due to the rigid design of the chitosan, the entanglement of SCHA expanded in Tween 20. Tween 20 was incorporated between chitosan layers, strengthening the chitosan NPs and increasing the ability of the medication to bind to molecules. Additionally, it increased particle strength and prevented the chitosan NPs from leaking.

Y3 = 14.46 + 0.9275 X1 + 0.9925 X2 + 0.5275 X3 + 0.1450 X1X2 − 0.4000 X1X3 − 0.9550 X2X3 + 2.37 X1^2^ + 0.7955 X2^2^ − 1.73 X3^2^

### 2.2. Point Prediction

**Figure 2:**
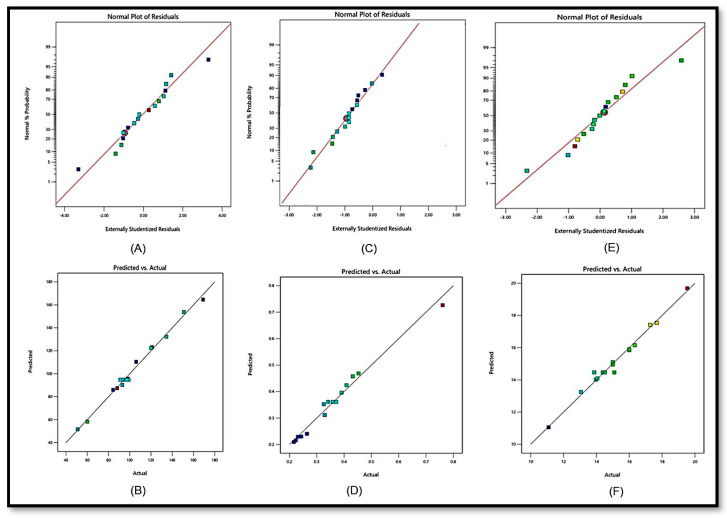
Actual and predicted graph of particle size (Y1) (**A**,**B**), polydispersity index (Y2) (**C**,**D**) and entrapment efficiency (Y3) (**E**,**F**).

**Figure 3:**
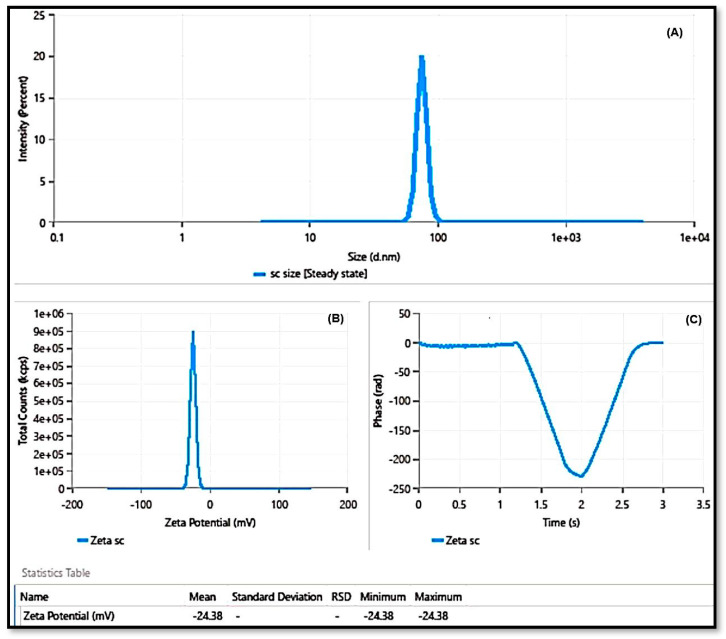
Particle size of SCHA-CS-NPs (**A**); zeta potential of SCHA extract-loaded chitosan nanoparticles (**B**); and phase–time plot of SCHA extract-loaded chitosan nanoparticles (**C**).

The best composition of SCHA-CS-NPs was chosen using the point prediction approach for further characterization. The optimized SCHA-CS-NPs were produced with chitosan (0.125 mg), Tween 20 (0.625 mL), and stirring speed (300 rpm) using the numerical point prediction optimization approach. The results were similar for all the dependent variables when using the improved composition. The agreement between the actual and predicted values of each dependent variable was interpreted using the graphs (Figure 2). A linear relationship was found between the value and the center. A particle size of 91.09 nm, an EE of 84.54%, and a PDI of 0.342 were observed. The particle size was 94.836 nm, the EE was 72%, and the PDI was 0.3611 according to the projected value (Figure 3). The average zeta size was 91.4 nm, and the PDI was 0.2636. The mean of peak 1 based on the intensity ordered area was calculated at 73.5 nm and 100%. The zeta potential was observed at −24.38 mV. The conductivity was 0.04891 mS/cm, and the wall zeta potential was −40.97 mV. The mean zeta deviation was 3.855 mV. The derived mean count rate was 8848 kcps, while the reference beam count rate was 1872 kcps. The quality factor was observed at 5.1.

### 2.3. FTIR

**Figure 4:**
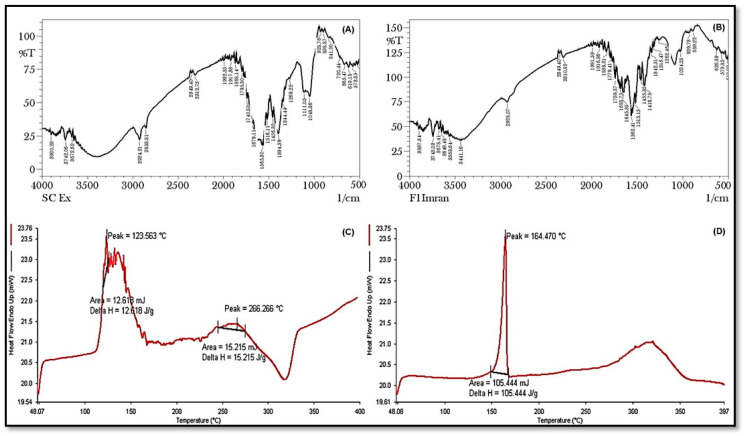
Fourier transform infrared spectroscopy (FTIR) spectra of the prepared SCHA extract (**A**) and SCHA-CS-NP (formulation) (**B**); differential scanning calorimetry (DSC) of the SCHA extract (**C**) and SCHA-CS-NPs (**D**).

FTIR spectra were frequently obtained to recognize and categorize biomolecules that may be suitable for capping, leading to the effective stability of the NPs. The NP-containing samples were thoroughly dried, crushed with KBr pellets and examined. Scan verification was conducted to obtain a suitable signal-to-noise ratio for the intended outcomes [12]. Stretching bands were observed above 1600 cm^−1^, and bending and stretching coexisted below this. A total of 15 bending and 12 stretching vibrations were discovered in the spectra of the SCHA extract (Figure 4A,B). When spectroscopy was used to analyze the SCHA-CS-NPs, the outcome remained consistent; the only change observed was the bond formed at 3100 cm^−1^. Similar studies have provided data including a comparison of peak values with corresponding wave numbers and potential functional groups identified during the FTIR analysis. Distinctive bands were observed in the aqueous extract of the research formulation, indicating N-CH_3_ out-of-phase bending at 1484.7 cm^−1^, N-CH_3_ in-phase stretching at 1414.9 cm^−1^, aryl-CH_3_ in-phase stretching at 1383.5 cm^−1^ and C-O stretching at 1086.6 cm^−1^. Conversely, in the alcoholic extract, characteristic peaks were noted, signifying -NH_2_ stretching at 3314.6 cm^−1^, -CH_2_ in-phase stretching at 2877.6 cm^−1^, (N)-CH_3_ in-phase stretching at 1418.4 cm^−1^, -CH_3_ in-phase bending at 1378.2 cm^−1^, aryl-N stretching at 1275.2 cm^−1^ and N-O stretching at 801.9 cm^−1^. This suggests that similar types of functional groups were found in both the aqueous and alcoholic extracts of the research formulation, implying the presence of amides, aldehydes, alkaloids and phenolic compounds in these extracts [13].

### 2.4. DSC

DSC rapidly and accurately determines whether a drug(s) and its excipients are compatible with each other, giving clear details regarding potential interactions. DSC thermograms of the drug’s formulations and pure drugs were obtained. The melting point of the SCHA extract was recorded as 123.563 °C, while the melting point of SCHA-CS-NPs was recorded as 164.470 °C. The melting peak of the SCHA extract was absent, as observed in the DSC spectra of the preparation, confirming the encapsulation of the extract in the NPs (Figure 4C,D).

### 2.5. TEM

**Figure 5:**
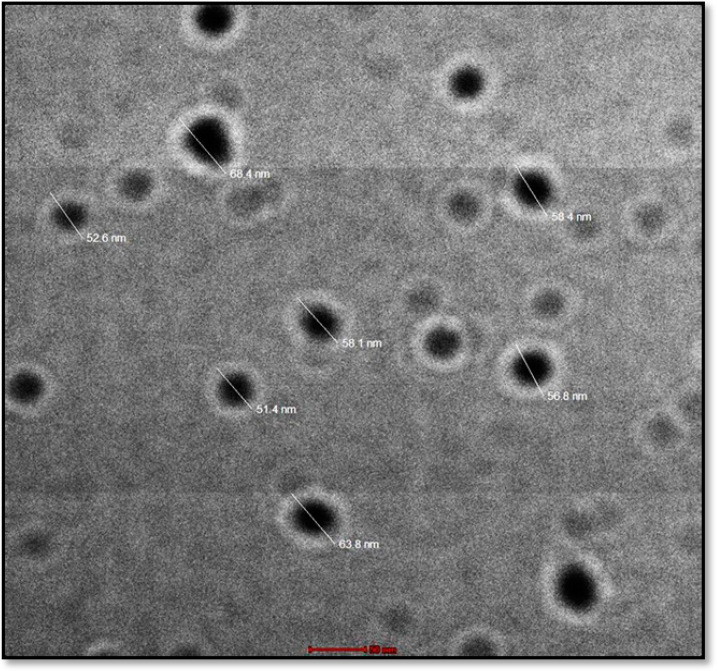
Transmission electron microscopy image of the produced formulation.

The morphology of the SCHA-CS-NP was analyzed using TEM. The results showed spherical structures of size 57.4 nm, consistent with the DLS results (Figure 5). The size of the NPs was 5 nm, as observed in previous studies on green synthesized NPs using *Sida cordifolia* plant extract in its aqueous form [14]. Similarly, another study reported an asymmetrical NP structure with an average size of 16 nm when α-Fe_2_O_3_ NPs were prepared using *Sida cordifolia* extract [15]. However, when silver NPs were prepared with *Sida cordifolia* extract, the TEM images revealed sizes from 10 to 30 nm [16]. Notably, all these studies used *Sida cordifolia* in its extract form with other compounds; therefore, the sizes may have varied.

### 2.6. In Vitro Release Study

**Figure 6:**
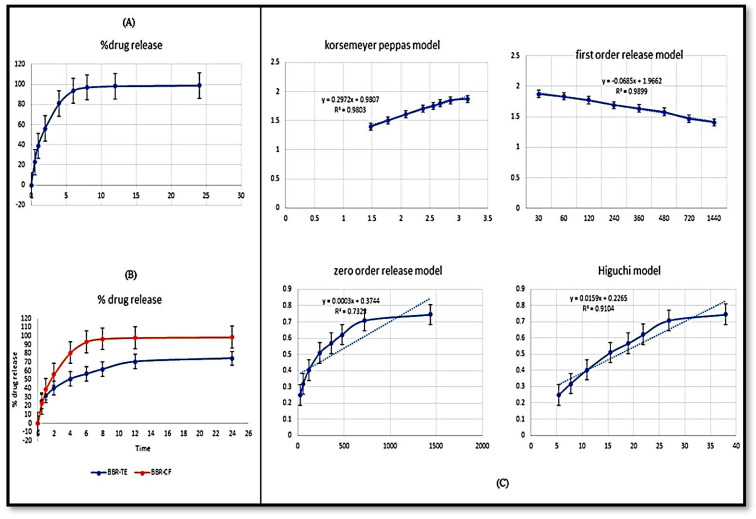
In vitro release of SCHA extract suspension (**A**); a comparative in vitro release profile of SCHA-CS-NPs and SCHA suspension was performed in triplicate, and data are shown as mean + SD (**B**). Study models were used for calculating the release of the drug (**C**).

The release behavior of the SCHA-CS-NPs and SCHA suspension was investigated to assess the release of the SCHA extract (Figure 6). The findings of the release analysis showed a maximum release of plain SCHA of 75% at 12 h compared to SCHA-CS-NPs, which released a maximum of 95% SCHA in 12 h. Only a 95% release was shown by the SCHA-CS-NPs at 12 h. At each time point, a tunable release was accomplished. In comparison to the SCHA suspension, the produced SCHA-CS-NPs demonstrated extended drug release due to encapsulation in chitosan NPs, causing slow diffusion to prolong the release pattern.

Figure 6 indicates that the first 2 h burst of drug release was followed by 24 h of slower drug release. This release behavior was excellent for improved treatment efficacy. Initial rapid release aids in achieving a therapeutic concentration, while extended slower release aids in achieving a superior therapeutic impact. The SCHA and SCHA-CS-NPs produced 98.06% and 70.72% release, respectively. It was established that the difference in the first 2 h was statistically significant (*p* ≤ 0.05). The in vitro release experimental data were fitted to various models (Figure 6).

A specific model was chosen for the study after testing several different models for forecasting drug release and analyzing the outcomes. Four different models, the Korsmeyer–Peppas, first-order, zero-order, and Higuchi models, were combined. According to the results of the various models, the Korsmeyer–Peppas model was followed to complete the study, as it had the highest R^2^ value.

### 2.7. CLSM Visualization

**Figure 7:**
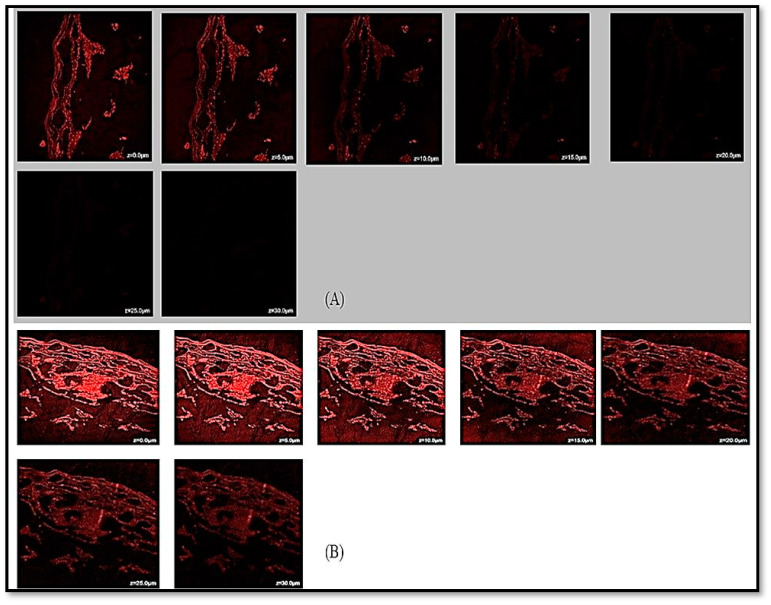
Confocal laser scanning microscopy (CLSM) image of rat intestine tissue treated with rhodamine B solution showing the depth of penetration (**A**) and rat intestine tissue treated with rhodamine-loaded SCHA-CS-NPs showing the depth of penetration (**B**).

The penetration depth of the tested substances was shown based on the comparative CLSM investigation results. The results of the CLSM analysis showed that, compared to the rhodamine B solution, rhodamine-SCHA-CS-NPs were highly permeated through the various layers of the intestine (Figure 7). Unlike the rhodamine solution, the results showed that rhodamine-SCHA-CS-NPs (Figure 7) were more equally distributed throughout the tissue. The distinct intracellular compartments that were darkened with the rhodamine demonstrated that transcellular transport accounted for most of the increased permeability. The SCHA-CS-NPs extended the contact time with the intestinal mucosa, which was determined as the cause of the improved permeability. For NPs loaded with the SCHA extract, the enhancement ratio was 1.25 times greater. SCHA has greater permeability due to its higher lipophilicity [17]. The size is significant in determining how well the medicine permeates and how easily it is absorbed. The SCHA-CS-NP showed the penetration of NPs in intestine to be 30 µm, while the suspension showed a penetration of up to 20 µm. This enables the penetration of particles smaller than 200 nm, and the generated SCHA-CS-NPs displayed a size smaller than 151 nm [18]. 

### 2.8. Antioxidant and α-Amylase Assay

The biological activity of plant-based bioactive chemicals is greatly influenced by antioxidant activity. The antioxidant activity was assessed using DPPH. It is important to assess the activity to determine whether excipients interfere with the antioxidant properties after formulation. The synthesized SCHA-CS-NP antioxidant potential was assessed and contrasted with those of the pure SCHA and common ascorbic acid. The enhanced potential of the SCHA-CS-NPs was confirmed by the stronger antioxidant value. Ascorbic acid and the SCHA-CS-NPs exhibited significantly different activity levels; however, the difference was not statistically significant (Table 4). The results of the α-amylase assay as depicted in Table 5 presents the findings of the antidiabetic potential produced by the prepared nanoformulation.

## 3. Materials and Methods

### 3.1. Materials

All the chemicals, reagents, chitosan (CAS No. 9012-76-4), Tween20 (CAS No. 9005-64-5) and solvents were purchased from Merck Sigma-Aldrich (Burlington, MA, USA). The aerial part of the species of the plant *Sida cordifolia* was collected from Varuni Export, Chennai (India).

### 3.2. Extraction

The aerial part of *Sida cordifolia* was dried, crushed into powder form and retained in airtight bags at approximately −80 °C. The extraction was performed using the Soxhlet method using water/ethanol (70:30) and distilled water as the solvent solutions.

### 3.3. Optimization and Development of NPs

SCHA-CS-NPs were optimized using the BBD, and three parameters were considered. The optimization was achieved using Design-Expert software. The impacts of various operation variables (chitosan concentration (X1), Tween 20 concentration (X2) and stirring speed (X3)) on the particle size (Y1), PDI (Y2) and EE (Y3) of the NPs were comprehensively examined. These independent factors were measured at low (-), medium (0) and high (+) levels to obtain the best composition. The design included 17 formulation runs of various compositions with three central points to investigate the effects of the independent variables. Polynomial equations and response surface plots were used to assess the effects of independent variables. Different models, such as the linear and quadratic effects of independent factors on dependent variables, are given by the polynomial equation. The optimal model among the various models is quadratic, since the variables used revealed individual and combined effects on the dependent variables (Table 1) [12].

### 3.4. Preparation of SCHA-CS-NPs

The SCHA-CS-NPs were prepared using an ionotropic gelation method. Chitosan was initially dissolved in diluted acetic acid solution and stirred overnight to acquire a homogenous solution. After adding Tween 80, the herbal extract was weighed, dissolved in a suitable solvent and added dropwise to the chitosan solution. The mixture was stirred for 2 h. The nanometric structures were obtained by adding sodium tripolyphosphate (TPP) at a feed rate of 1 mL/h and maintaining a stirring speed of 600 rpm until an opaque solution was obtained, indicating the formation of NPs, as described in [10]. The SCHA-CS-NPs were encapsulated in two steps [19].

Emulsification of the oil in the water was followed by modified ionic gelation. Chitosan was dissolved in 1% *v*/*v* acetic acid using a 60 min sonication process to produce chitosan solutions at 1% *w*/*v*. Subsequently, this solution was filtered through a 1 µm particle filter to remove any remaining undissolved chitosan. A total of 50 mL of chitosan solution was mixed with 80 mg of Tween 80 before raising its pH to 4.2 using 2 N NaOH solutions.

The liquid was agitated at 50 °C for 90 min to produce a homogeneous solution. Different amounts of *Sida cordifolia* extract were added to this mixture to obtain different ratios.

*Sida cordifolia* extract was continuously poured into 50 mL of chitosan aqueous solution during homogenization at 13,000 rpm for 10 min to create an oil-in-water emulsion. Sodium TPP was added last to allow the chitosan to gel ionically. The solution was continuously agitated for 40 min. The former particles were separated using centrifugation at 9000× *g* at 4 °C for 30 min, and they were then repeatedly rinsed with deionized water. These suspensions were promptly freeze-dried for 42 h at −32 °C. The prepared NPs were characterized further using FTIR, DSC, a Zetasizer, TEM and other techniques.

### 3.5. Characterization of SCHA-CS-NPs

#### 3.5.1. FTIR Analysis

A FTIR analysis was conducted to verify the production of NPs. Each sample for the FTIR was produced as a pellet in potassium bromide (KBr) at a ratio of 1:99, and measurements were made with a resolution limit of 16 cm^−1^ [17].

#### 3.5.2. DSC Analysis

DSC is a thermodynamic method for directly evaluating the heat energy uptake occurring in a sample during a controlled temperature increase or reduction. Particularly, calorimetry is used to track changes in phase transitions [20]. DSC shows that melting temperatures vary according to grain size. Due to a higher surface-to-volume ratio, the melting temperature of NPs can be 10 to 100 K lower than that of the bulk material. SCHA extract and SCHA-CS-NPs were characterized using DSC following a proper protocol and values.

#### 3.5.3. Particle Size and PDI Determination

A particle size analyzer was used to evaluate the particle size, PDI and zeta potential of the generated SCHA-CS-NPs. The surface charge was calculated using the zeta potential, and it was constant at +30. The samples were diluted 100 times in double-distilled water before being filtered through a 0.45 µm membrane filter and examined.

#### 3.5.4. EE (%)

An ultracentrifugation filtration technique was performed to measure the EE of the SCHA-CS-NPs [21]. These samples were ultracentrifuged (Beckman Coulter; LE 80) at 25,000 rpm for 30 min at 4 °C [14]. After collecting the supernatant, the free drug was measured using a spectrometric detection technique at 291 nm. Equation (1) was used to determine the EE.

% EE = D_int_ − D_sup_./D_int_. × 100 (1)

D_sup_ is the quantity of SCHA found in the supernatant, and D_int_ is the detected amount of SCHA in SCHA-CS-NPs.

#### 3.5.5. TEM

The morphological review was performed using TEM (TEM-Tecnai, G20, Philips logical, and The Netherlands). Briefly, the NPs were diluted to obtain an aqueous solution. A drop of the dispersed solution was then placed on a copper grid and allowed to dry. The morphology of the preparation was then examined at a magnification of 80,000×.

#### 3.5.6. Drug Release Study

Dynamic dialysis is one of the most well-known techniques for determining the release kinetics from NP drug delivery systems. A dialysis-based in vitro release study was conducted to examine the release of NPs loaded with SCHA extract. A pre-treated dialysis bag (MW—12 kDa) was filled with SCHA-CS-NPs (5 mg SCHA), and both ends were knotted. The dialysis bag containing the samples was submerged in a 250 mL saline buffer solution with a pH of 7.4 and 25% methanol. The beaker was maintained on a magnetic stirrer at 300 rpm and 37 °C using a thermostatic control. The discharged samples (2 mL) were collected and replaced at various intervals with the same volume (up to 24 h). The discharged material underwent spectrophotometric analysis at 291 nm [22].

#### 3.5.7. CLSM Analysis

A confocal examination was conducted to compare the penetration depths of the synthesized SCHA-CS-NPs into the rhodamine solution. Rhodamine B red dye-stacked SCHA-CS-NPs were developed to measure the penetration depth. The experiment was conducted similarly to the permeation study. The rhodamine-loaded NPs and rhodamine solution were exposed to the fixed intestine cells via diffusion. The rhodamine solution was used as a control sample. After 6 h, the treated mouse intestine was removed, cleaned with double-distilled water and cut up to make a microscopic slide. The slides were analyzed using CLSM to monitor the particle penetration into the intestinal layers. Rhodamine B was optically stimulated in this study using a 488 nm argon laser beam, and the fluorescence emission extending past 532 nm was analyzed. The depth of NP penetration was assessed using CLSM in comparison to the control. The permeation study included rat intestinal tissue. Normal saline buffer was used to store the separated cells after cleaning them twice or three times with distilled water (pH 6.4). A diffusion cell was used to study the permeation investigations; this had donor and receiver compartments and an effective surface area of 1.5 cm^2^. The intestinal cells were fixed when the cell surface was directed toward the receiver compartment [23]. The receiver chamber was filled with 10 mL of phosphate buffer solution (pH 6.4), including 7% propylene glycol and 25% methanol. The temperature was maintained at 37 °C to approximately match the temperature of the human body. A magnetic stirrer was attached to the diffusion cell, and stirring was maintained at 100 rpm. Two milliliters of SCHA solution and a sample of NPs loaded with SCHA extract were placed inside the donor cell. Aliquots (1 mL) were periodically removed from the receiver compartment and replaced with a new release medium to maintain the study conditions. The removed samples were subjected to spectrophotometric evaluation at 291 nm. Each experiment was repeated three times to produce the average findings [24].

### 3.6. Antioxidant Assay

The antioxidant potential of SCHA-CS-NPs was assessed using the DPPH method. A common technique for determining a compound’s antioxidant potential is the DPPH free radical approach. At normal temperatures, the violet color of the DPPH solution disappears due to the antioxidants’ capacity to transfer electrons [16]. Before using the DPPH methanolic solution on the sample (0.5 mL), 3 mL of methanol was used to dissolve it (0.3 mL). The reaction mixture was kept in a dark area for 1 h to complete the reaction process. Due to the sample’s ability to donate hydrogen, the color change reflected its antioxidant capacity.

The blank contained 3.3 mL of methanol and 0.3 mL of sample, whereas the control contained 3.5 mL of methanol and DPPH solution (0.3 mL). Spectrophotometry was employed to analyze the substance at 517 nm.

### 3.7. α-Amylase Assay

An α-amylase assay was performed to observe the antidiabetic potential of the prepared nanoformulation. Starch azure was introduced as a substrate as a 0.5% starch solution after the α-amylase (0.5 mg/mL) was premixed with extract at several concentrations (100–500 g/mL) to start the reaction. After 10 min at 37 °C, the reaction was stopped by adding 23 mL of the 3, 5-dinitrosalicylic acid reagent. After heating for 20 min at 100 °C, the reaction mixture was diluted with 15 mL of distilled water in an ice bath [25].

A spectrophotometer was used to measure the activity of α-amylase at 540 nm. The test sample was not used in the control reaction. The trials were conducted three times using the same methodology. The percentage of the α-amylase inhibitory activity was calculated using the following formula:

$$(\%\;\mathrm{of}\;\mathrm{Inhibition})\;=\;\frac{Control_{Optical\;Density}-\;Sample_{Optical\;Density}}{Control_{Optical\;Density}}\times 100$$

### 3.8. Statistical Analysis

The statistical analysis of the data was verified using GraphPad Prism (v 5.0, GraphPad Software Inc., San Diego, CA, USA), and one-way analysis of variance (ANOVA) procedures were used. The mean and standard deviation were used to summarize the results [26].

## 4. Conclusions

Numerous tests were conducted to characterize and improve the developed formulations. By establishing the ideal values for NP preparation, the BBD was applied to maximize the creation of the NPs. The actual and projected values of the findings were characterized using the point prediction approach. The effective stability of the produced NPs was assessed using FTIR. Consequently, 15 bending and 12 stretching vibrations were observed using spectroscopy. The drug’s melting point was obtained as 125.563 °C using DSC. When the size of the synthesized NPs was examined using TEM, a consistent value of 57.4 nm was obtained. Various models were used to calculate the in vitro drug release from the NPs. The Korsmeyer–Peppas model was used to complete the study, as it had the highest R^2^ value. The degree of drug penetration was assessed using confocal microscopy. The outcomes demonstrated that the SCHA extract-loaded solutions had a high level of penetration into the intestinal layer. The NP-based drug delivery system is branching into the encapsulation of herbal extracts to improve their efficacy. The BBD was employed with the formulations to obtain the best nanopreparation. The effects of three independent variables (chitosan and Tween 20 concentrations and stirring speed) on particle size, encapsulation efficiency and PDI were studied. Furthermore, the best formulation was characterized. The developed nanoformulation showed an extended-release profile due to encapsulation inside the NPs. Moreover, compared to plain rhodamine-bound SCHA extract, the NPs had better penetration, which is required for synergistic extract effectiveness. A noticeable antioxidant activity was observed for SCHA-CS-NPs due to the flavonoid and phenolic content present in the SCHA extract. Overall, the results demonstrated that SCHA-CS-NPs could be a promising carrier for the effective uptake of SCHA extract in treating diabetes mellitus.

## Figures and Tables

**Table 1:** Level of independent variables used to optimize *Sida cordifolia* hydroalcoholic (SCHA) extract-loaded chitosan nanoparticles (CS-NPs) using the Box–Behnken design (BBD).

Variables	Low	Medium	High
Independent variables			
X1 Chitosan (%)	0.05	0.125	0.2
X2 Tween 20 (%)	0.25	0.625	1
X3 Stirring speed (Rpm)	200	300	400
Dependent variables			
Y1 Particle size (Nm)			
Y2 Polydispersity index (%)			
Y3 Entrapment efficiency (%)			

**Table 2:** The observed response value in BBD for optimization of SCHA extract-loaded chitosan nanoparticle (SCHA-CS-NP) formulations created using Design-Expert software.

Runs		Independent Variables			Dependent Variables		
	Formulation	Chitosan (%) (X1)	Tween 20 (%) (X2)	Stirring Speed (rpm) (X3)	Particle Size (nm) (Y1)	Polydispersity Index (Y2)	Entrapment Efficiency (%) (Y3)
1	F1	0.125	0.625	300	91.09	0.342	78.21
2	F2	0.125	1	200	151	0.432	74
3	F3	0.2	0.625	400	106	0.231	81.23
4	F4	0.125	0.625	300	94	0.359	73.9
5	F5	0.05	0.25	300	121	0.243	80.21
6	F6	0.2	1	300	134.47	0.391	84.54
7	F7	0.2	0.625	2000	88.06	0.761	79.25
8	F8	0.125	0.25	400	93	0.329	65
9	F9	0.125	0.625	300	97.09	0.359	67.89
10	F10	0.05	0.625	400	51	0.326	75.54
11	F11	0.125	0.625	300	99	0.371	72
12	F12	0.2	0.25	300	60.03	0.453	79.09
13	F13	0.05	0.625	200	169	0.216	55.90
14	F14	0.125	1	400	98	0.223	61.89
15	F15	0.05	1	300	84.31	0.264	80.07
16	F16	0.125	0.625	300	93	0.359	56
17	F17	0.125	0.25	200	120.08	0.409	44.76

**Table 3:** Summary of parameters for response Y1 (particle size), Y2 (polydispersity index) and Y3 (entrapment efficiency).

Responses	R^2^	Adjusted R^2^	Predicted R^2^	SD	% CV	Model
Y1 (nm)	0.9914	0.9804	0.9046	4.13	4.01	Quadratic
Y2	0.9822	0.9643	0.8371	0.0240	6.74	Quadratic
Y3 (%)	0.9844	0.9644	0.9327	0.3644	2.41	Quadratic

**Table 4:** Antioxidant assay of SCHA and SCHA-CS-NPs.

S. No.	Name of the Drug	IC_50_ (µg/mL)
1	SCHA extract	82.36 ± 1.31
2	SCHA-CS-NPs	86.45 ± 2.24
3	Ascorbic acid (standard)	88.23 ± 1.43

**Table 5:** α- amylase assay of SCHA and SCHA-CS-NPs.

S. No.	α-Amylase Activity	IC_50_ (µg/mL)
1	SCHA extract	90.23 ± 1.64
2	SCHA-CS-NPs	93.71 ± 1.79
3	Acarbose (standard)	97.25 ± 1.43

## Data Availability

The data will be available from the authors upon reasonable request.
